# Growing Up: Not an Easy Transition—Perspectives of Patients and Parents regarding Transfer from a Pediatric Liver Transplant Center to Adult Care

**DOI:** 10.1155/2015/765957

**Published:** 2015-11-22

**Authors:** Sona Chandra, Shannon Luetkemeyer, Rene Romero, Nitika Arora Gupta

**Affiliations:** ^1^Department of Biomedical Engineering, Georgia Institute of Technology, Atlanta, GA 30332, USA; ^2^Children's Healthcare of Atlanta, Transplant Services, Atlanta, GA 30322, USA; ^3^Department of Pediatrics, Emory University School of Medicine, Health Science Research Building (HSRB), Suite No. E200, Office No. 216, Atlanta, GA 30322, USA

## Abstract

The transition from pediatric to adult care is a critical time when children with chronic illness sustain high morbidity and mortality. Transition services need to be focused on the adolescents' needs, which may differ from those perceived by healthcare providers. In this study, a survey of 31 patients with chronic liver disease and/or liver transplant who were “transferred” to adult services within the last 3 years was conducted. Patients were asked about their current health status and their perceptions of the overall transfer process. The mean age at transfer was 19.81 (18–21) years. Almost half the patients (47%) were not seen at the adult facility until 2–6 months after leaving the Children's hospital and 20% were not seen until 6–12 months. About 20% had their first contact with adult services through an emergency room visit. About 19% reported being out of medication during transition. Of the transplanted patients, 19% were being evaluated for a retransplant. The majority (82%) felt that an increased emphasis on promoting independence and “letting go” both by parents and by pediatric care providers was critical in their transition to independence and adult care services. This study provides thought-provoking insights, which are critical in designing the ideal transition program for children with chronic diseases.

## 1. Introduction

With improved outcomes of postliver transplantation, a large number of pediatric liver transplant recipients are reaching adulthood [1]. The posttransplantation five-year graft/survival rate for pediatric liver transplant recipients currently ranges from 67% to 82% [2, 3]. Therefore, the need to prepare pediatric liver transplant recipients for the transfer to adult-centered transplant care has increasingly become an important area of investigation.

According to the American Society for Adolescent Medicine, the transfer process should include a planned movement of adolescents with chronic medical conditions that addresses their medical as well as psychological needs as they shift from pediatric to adult-oriented healthcare systems [4, 5]. There are a number of barriers that a young adult transplant patient faces, with the most important one being the development of a sense of autonomy and independence [6]. The suggested level of autonomy before transferring to adult care includes the patient's adequate demonstration of knowledge of the disease and transplant as well as a thorough understanding of the impact on his or her overall health [2]. In addition, the patient should be able to complete his or her medication regimen, refill medications independently, make and attend appointments, and recognize how and when to seek medical attention [7]. Furthermore, avoiding risky behaviors such as poor diet, unsafe sexual activity, drug and alcohol abuse, and bad hygiene is essential to the success of a patient [8]. A fully independent, self-governing, and autonomous adult patient at the time of transfer is a common goal of pediatric healthcare providers.

Patients who have undergone liver transplantation in their childhood are more likely to display a delayed sense of independence and are at risk for medication nonadherence [2]. A recent study revealed that nonadherence ranged from 17% to 53% [9, 10]. Nonadherence can have severe consequences including poor graft survival rates, increased need for clinical visits, and rejection episodes [7].

As pediatric patients approach the age for the transfer to adult care, it is increasingly important for healthcare professionals to effectively equip their adolescent patients with the knowledge and support to cope with the transfer. This study is aimed at assessing the patient/parent perspectives of the transfer process 1–3 years after their transfer to adult care in order to develop a more effective transition program. In addition, the survey asked for suggestions for the improvement of facilities and the changes pediatric caregivers can make to facilitate the above. This information can help caregivers and healthcare providers shape their services to better target patient needs and ultimately encourage independence and autonomy amongst adult patients.

## 2. Materials and Methods

### 2.1. Patients and Methods

Transplant patients, who have previously been transferred from pediatric care at Children's Healthcare of Atlanta to adult care at various different facilities, completed a survey designed to assess the issues they faced during the transition and their suggestions regarding the transfer process. This study was conducted prior to the establishment of a formal transition program at Children's Healthcare of Atlanta and was aimed at patients who had undergone a “simple transfer” process from Children's Healthcare of Atlanta to adult services in the past 3 years. The study was performed as a quality improvement service and data were analyzed retrospectively. It was reviewed and approved for an exemption by the Institutional Review Board of Emory University. All patients who were between 1 and 3 years after transfer to the adult facility were contacted for a survey over the phone. If the patients were unavailable, the survey was conducted with the parents, only if the parents were involved with the care of the child and accompanied them to clinic visits at the Children's hospital. Patients who had been diagnosed with chronic liver disease but had not undergone a transplant were included in this study because they were followed by the same medical team hence facing a similar “transfer” process. Additionally, the challenges faced during the transfer to adult care were very similar to the challenges faced by transplant patients [11]. A nurse navigator who was familiar with the patients and had been closely involved with their care while in the pediatric facility conducted the survey.

### 2.2. Survey

The survey was designed to assess the effectiveness of each patient's transfer via certain key markers of health outcome such as (i) ER visits; (ii) time until the patient's first labs are drawn in the adult care; (iii) liver function tests; (iv) number of rejection episodes; and (v) the evaluation for a new transplant. In addition, the survey asked for any suggestions these patients or parents may have had regarding the transition process such as (vi) additional education on the part of healthcare professionals, (vii) parent involvement and management of their children, and (viii) changes that could be implemented to evolve the current transition process.

Phone survey respondents were primarily given questions with multiple choice answers assessing the basics of their transfer process including, for example, how much medication they were given at the time of transfer as well as the number of emergency room episodes that occurred in the interim period. The respondent additionally had the option of submitting an alternate, “free-form” answer that was not listed for each question. There were three questions which assessed the patient's transfer experience and any suggestions that respondents may have regarding the transfer process (Tables 1–3). The suggestion part of the survey gave several different options for each question so that each person surveyed was able to choose the answer(s) that best reflected their perspective on the transition. The patient or parent surveyed additionally had the option of inputting their own suggestion if it was not listed.

The survey included questions about the patient's medication access and availability for refill because they are important indications of the effectiveness of the transfer. Adherence to immunosuppressant medications is a critical factor in the transfer process as poor medication adherence is associated with an increased risk of poor long-term health outcomes, including increased graft loss and rejection [10, 12].

## 3. Results

### 3.1. Patient Demographics

All patients were within the range of 18–23 years (median age: 20). Majority of them were Caucasians (57%), with 33% being African American and 10% being Hispanic. Females were predominant in this group (70%). Of the 31 survey respondents, 39% were patients, and the remaining 61% were parents of patients. There were 4 patients (or parents) who did not have a working phone number and were unable to be contacted. Hence, our response rate was 31/35 (88.5%).

### 3.2. Patient Diagnosis and Transplant Information

**Figure 1:**
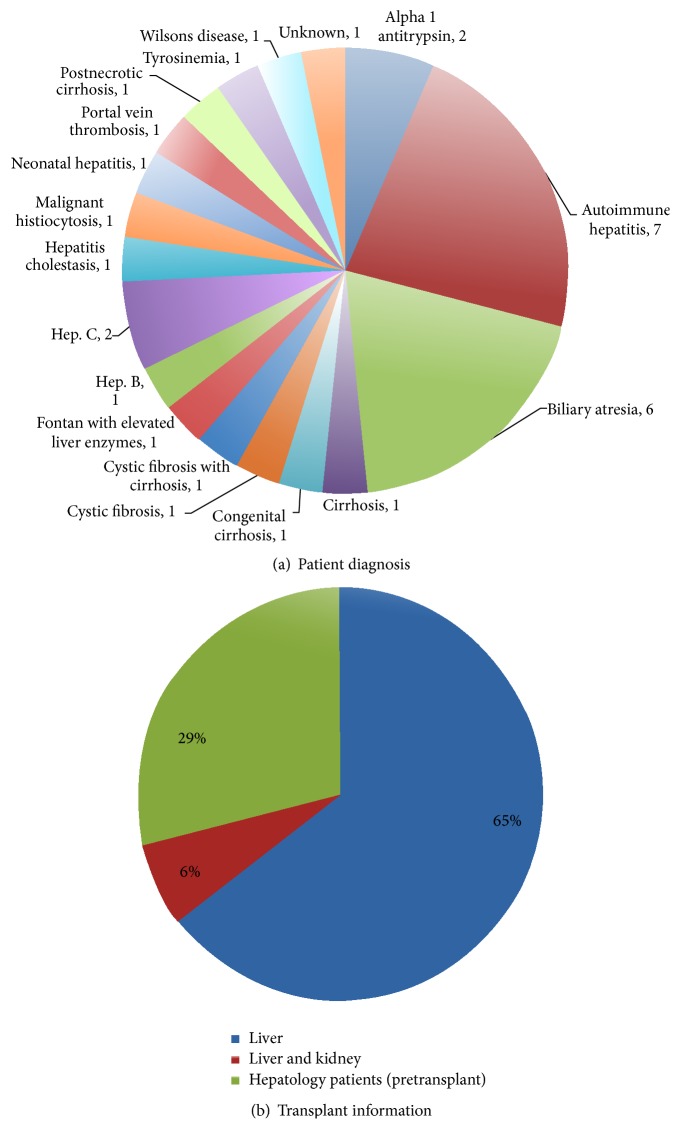
Patient diagnosis and transplant information. (a) Patient diagnoses were distributed over a large spectrum and were representative of the larger population of children requiring a liver transplant. (b) Distribution of patients with isolated liver transplantation, liver-kidney combined transplantation, and preliver transplantation.

The primary diagnosis for liver transplant and chronic liver disease was reported by the patients and parents and is detailed in Figure 1(a). The largest percentage of surveyed patients had autoimmune hepatitis with the second largest group being biliary atresia. The majority of the patients surveyed had received a liver transplant (67%) with two (6%) receiving both a liver and kidney transplant. The rest of the patients had chronic liver disease of varying etiology (27%) and had not been evaluated for a transplant at the time of transfer (Figure 1(b)).

### 3.3. Time Lag between the Last Visit with the Children's Hospital and First Visit with the Adult Facility

**Figure 2:**
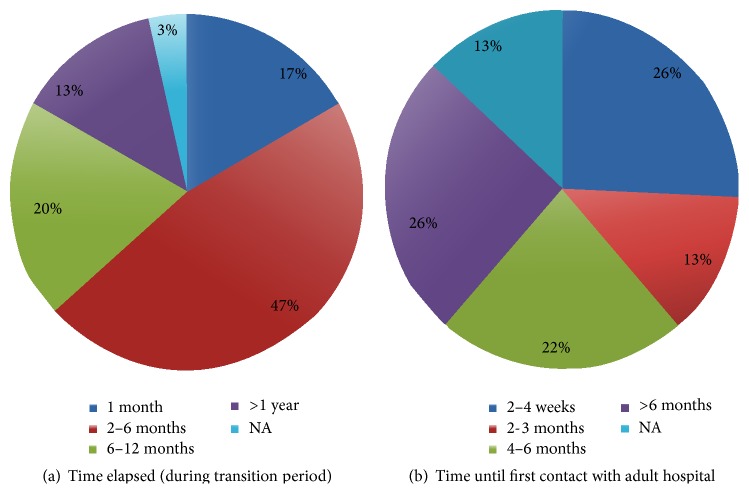
Time lag between the last visit with the Children's hospital and first visit with the adult facility. (a) Time between the last visit at the Children's hospital and the first visit with the adult care facility. (b) Time until first contact with Emory University to make/confirm a clinic appointment.

The time elapsed between the last visit at the Children's hospital and the first visit at the adult facility is an important predictor of nonadherence with clinic visits and the possibility of being lost to follow-up [10, 12]. At the time of the survey, it was our center's practice to recommend follow-up with the adult practice within 3 months of transfer from Children's Healthcare of Atlanta. A small number of young adults (17%) were seen within one month of leaving the Children's hospital facilities. The majority of patients (47%) were seen at the new facility 2–6 months after their last scheduled appointment with the Children's hospital. Furthermore, about 20% of the young adults were not seen until between 6 and 12 months and 13% were not seen until after a year (Figure 2(a)). This was regardless of the adult facility they were transferred to.

We also assessed the time until the new facility was first contacted to make/confirm an initial appointment after transfer because this is reflective of the efficiency of the transfer, with 26% of patients contacting between 2 and 4 weeks, 13% between 2 and 3 months, 22% of patients between 4 and 6 months, and 25% not contacting until after six months. Some patients (13%) did not contact the adult facility to make an appointment.

### 3.4. Medication Supply at the Time of Transfer

**Figure 3:**
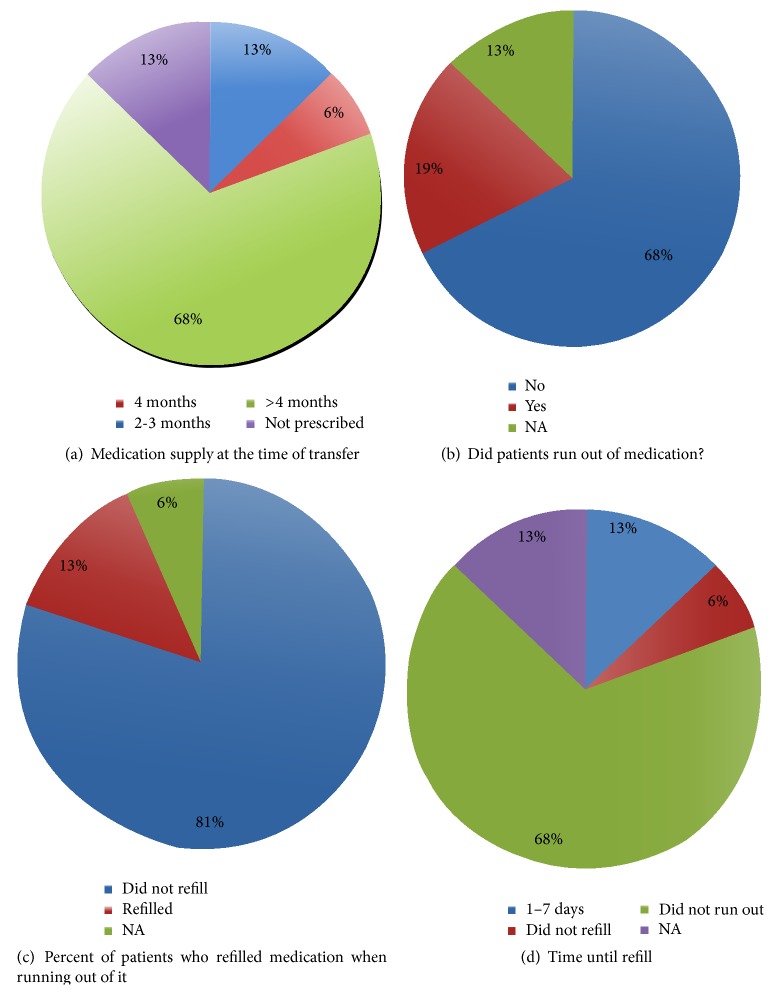
Medication supply at the time of transfer. (a) Amount of medication obtained at the time of transfer. (b) Patients who ran out of medications prior to being seen by the adult care facility. (c) Number of patients who refilled their medication prior to being seen by the adult care facility. (d) Time lag between refills.

Through our survey, we wanted to gain insight regarding the patients' adherence to their prescribed medication regimen. In order to accomplish this, we asked direct questions relating to the amount of medication they had at the time of transfer (Figure 3(a)). Interestingly, most patients (68%) had more than 4 months' supply of medication at the time of transfer, with 6.4% having just under 4 months' worth and 9.7% having 2-3 months' supply of medications (Figure 3(a)). This, however, could possibly be a reflection of the pediatric transplant center's prescribing practices rather than the patients themselves. Prior to seeing the adult caregivers, 68% of patients did not run out of medications. Some of the surveyed patients (19%) did run out of medication. The remaining 13% were unable to answer questions or were unsure of the amount of medication in their possession at the time of transfer (Figure 3(b)).

A patient's ability to refill their medication as needed is highly indicative of their autonomy. A majority of the patients (81%) reported that they did not need to refill their medication (Figure 3(c)). The majority of the patients did not run out of their medications (Figure 3(d)). Of the patients who did run out of medication, 67% were able to refill their prescription at some location (50% at the Children's hospital pharmacy, 25% at ER, and 25% other) within 1–7 days. The remaining 33%, representing 6% of the total patients, were not taking their medication.

### 3.5. First Contact with the Adult Facility

**Figure 4:**
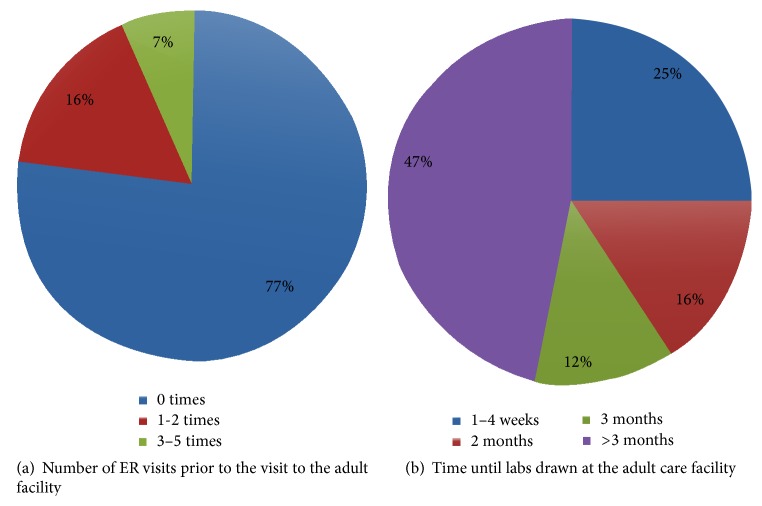
First contact with the adult facility. (a) Number of emergency room visits prior to being seen in the adult facility. (b) Time between last labs at the Children's hospital and when first drawn at the adult facility.

Having assessed the medication supply of the patients at the time of transfer and the time lag before they were seen at the adult facility, we wanted to evaluate the health status during the transition prior to first contact with the adult care providers. Interestingly, we found that 23% of the patients had a visit to the emergency room prior to seeing the adult care providers in the outpatient setting. Out of these, the majority (5/7; 77%) had 1-2 visits and 2/7 (29%) had 3–5 visits to the emergency room (Figure 4(a)). After leaving the Children's hospital, almost half of the patients (47%) did not get labs done until more than 3 months after transfer. Only a quarter of the patients reported labs being done within 1–4 weeks after transfer. The remaining 28% had their labs done between 2 and 3 months (Figure 4(b)).

### 3.6. Status of Liver Function

**Figure 5:**
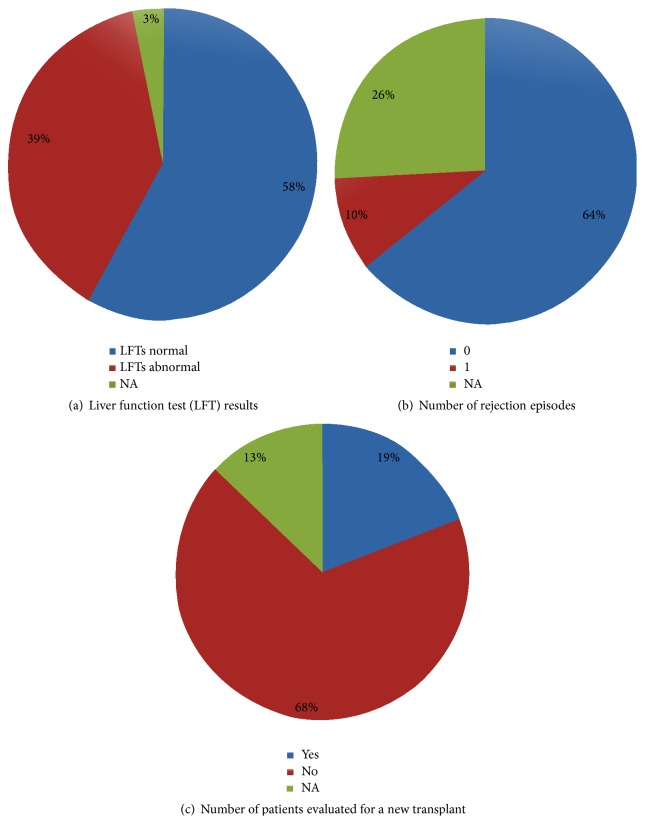
Status of liver function. (a) Status of the liver function tests. (b) Number of rejection episodes. (c) Number of patients evaluated for a new transplant.

While the timing of getting labs drawn was an important indicator of patient compliance or responsibility, it was also critical to assess the actual status of the disease or disease severity. At the time of the survey, more than one-third of the patients (39%) reported having abnormal liver function tests (Figure 5(a)), with 10% being diagnosed with acute rejection soon after being transferred from the pediatric facility. Most however had normal liver function and had not had a rejection episode (Figures 5(a) and 5(b)). About 19% of the patients were being evaluated for a new transplant. None of the patients with chronic liver disease were undergoing a transplant evaluation at the time of transfer (Figure 5(c)).

### 3.7. Patient and Parent Suggestions for Transition Clinics

At the time of this survey, the liver transplant service did not have a formal transition process. In order to establish an effective transition clinic, we wanted to assess patient needs and perspectives. We asked the patients who had transferred from the Children's Hospital to the adult services what they felt were the important components to make the transition to adult services smoother and of higher utility. More than half of the survey respondents were the parents. The difference between the suggestions and perceptions of patients and parents was striking. Education about the disease, transition process, insurance, and medications were all deemed extremely important by the parents but by only about quarter of the patients. About half of the patients did feel they needed to be educated about their disease with emphasis on the total body care (Table 1).

When asked about how parents and patients could be better prepared to ensure a smooth transition, a majority of the patients and the parents agreed on one thing: patients needed to be “let go” and empowered in order to assume self-care and independence. 25% of the patients felt that parents needed to be “less” involved in the care of the patients and very few of the parents and patients (10% and 16%, resp.) felt that there needed to be more involvement of the parents. About 50% of the patients felt that they should receive fewer reminders about their healthcare regimen and that the parent should trust them to be capable of self-care (Table 2).

Most parents felt that a parent support group would help them in learning how to empower their children and let them assume independent care, which can be incredibly difficult after several years of taking care of a child with a chronic illness (Table 2). The parents also reported that they would have preferred earlier education about the adult facilities, rather than information at the time of transfer.

It was interesting to note that one-third of the patients/parents felt that repeated reminders should not be given and were actually counterproductive to a good outcome. Of concern was the fact that there was a lack of interest reported from both the patients and parents with regard to education about insurance and what to do in the face of loss of insurance (Table 3).

## 4. Discussion

Liver transplantation is now an accepted treatment for several end stage liver diseases. The survival rate of patients with pediatric transplantation is at an all-time high as the posttransplantation five-year graft/survival rate for pediatric liver transplant recipients currently ranges from 67% to 82% [2, 3, 13, 14]. However, it is also clear by UNOS data that graft loss increases dramatically from ages of 16–30 years [15–17]. Since most pediatric and adult centers operate in their own freestanding hospitals, the process of transfer from pediatric to adult services has been deemed as one of the primary reasons for this graft loss [9, 10, 17]. Adolescents have not been prepared for or are unable to maneuver the demands of the adult healthcare system and hence find themselves lost in the maze leading to increased noncompliance, nonadherence, and eventual graft loss [18].

Our study set out to evaluate patient and parent perspectives of the transfer process in order to determine the key factors, which were critical to the establishment of a successful transition program. We wanted to assess this process from the patients' and parents' viewpoint, instead of exclusively basing it on what was deemed important by caregivers. This study provided us with some interesting perspectives, which enabled us to institute key components in the structure of our transition clinics, details of which are discussed below. These insights have applicability to all transition clinics catering to children with a wide variety of chronic diseases.

Most care providers worldwide attempt to provide enough medication refills to the patients prior to transfer [19]. In this study, we found that there was still a significant number who ran out of their medications and did not attempt to refill their prescription, which can be detrimental to the overall health outcomes. Similarly, since graft loss is high during this stressful time of transition, at the time of transfer, it was best to conduct more frequent labs, with the first labs and clinic visit being within one month of transfer, at the adult center [1]. Most of our patients did not make the first contact with the adult services until after 3 months of transfer, with a quarter of patients waiting more than 6–12 months, during which they had emergency room visits. This resulted in a “limbo period” in which the patients were technically “not owned” by any service and were not under the care of any provider, as is evident by the rejection episodes and graft dysfunction. This is a critical period and underlines the importance of instituting measures to bridge this gap.

In addition, clinic appointments for the adult care facility are best scheduled prior to transfer since a percentage of patients are likely to show up in the emergency room in critical condition. However, this does not always solve the problem, as it has been reported that patients do not show up for appointments even if they are scheduled beforehand [6, 9, 10, 20]. In order to alleviate some of the anxiety associated with the first visit, we have instituted joint clinics with the adult care providers with the goal that this will alleviate anxiety associated with the first visit and hence encourage the patient to be compliant and work towards building a relationship new care team.

Changes in medication prescription and scheduling of appointments and joint clinics with the adult care providers are structural issues that can be resolved by the medical providers. More difficult are the conceptual and ideological issues associated with pediatric medical teams, who have not only provided care to the children from sometimes as early as infancy and neonatal period, but have also bonded with them to the extent of being part of the “enabling team” along with their parents. The parents and patients both felt that there should be earlier teaching towards independent care and ownership. Neither the parents nor the providers followed the philosophy of “letting go.” One example of that was the repeated phone calls and reminders made by the pediatric care team for missed clinic and laboratory appointments.

Based on these recommendations, we changed the structure of a visit within our transition clinics to have a greater focus on self-empowerment and education about adult facilities. Instead of an entire care team seeing the patients during clinic, after the patient is 18 years old, the physician and the nurse saw patients exclusively, as occurs in adult clinics, without the parents being present. They were given the opportunity to voice their concerns and ask for any other care provider who they felt was necessary for them to see, such as the nutritionist, pharmacist, psychologist, and social worker. Updated “the parents” separately. For patients 14–16 years of age, the parents were asked to step out of the room for personal questions only. Between the ages of 16 and 18 years, the physician first saw the patients individually, and the appointment was concluded with the parent in the room. All patients were expected to make their own future appointments and given training to call in their own refills. The transition program was renamed Adolescent Program, “I own It” (AP 101), which emphasized self-care, empowerment, education, and independence.

We also instituted parent support groups, which included sessions on insurance, college courses, and jobs with benefits. This education was also provided to the patients, since it was concerning to see that very few felt education about their insurance was an important issue in the United States, where healthcare is not government provided [21].

As with any retrospective data collection, there were some limitations to this study. Markers of health outcomes (LFTs, rejection episodes, new transplant evaluation, etc.) as well as indicators of adherence, such as medication supply at the time of transfer and the first visit at the adult facility, were based on the responses of patients or parents. This method of data collection is subject to such limitations as lapses in memory or recall bias, which may have resulted in relatively inaccurate responses. While ideally a patient's health outcomes could be drawn from medical records, in this case it was extremely difficult to obtain access to those records considering that this study included patients who had been transferred to a number of different healthcare facilities both within and outside of the state of Georgia. As a result, we chose to rely on the responses given by the patient or parent. Furthermore, there could be a difference in the accuracy of information provided by a parent or a patient. However, we felt that this gave us a different perspective which also added valuable information.

The study group we chose was limited by its size of 31 survey subjects composed of 12 patients and 18 parents of patients, none of which were patient/parent dyads. We chose a cut-off of 1–3 years since the time of transfer in order to limit the amount of recall bias in the responses. Furthermore, we limited the study to liver transplant recipients and chronic liver disease patients since the same care team managed both, and the education provided to each patient was similar. We could have increased our sample size by introducing kidney and heart transplant patients but chose not to do so to avoid introduction of individual provider and care team practice styles, which may have skewed the data. Additionally, since the outcomes and longevity of the grafts between the solid organs are different [22], we wanted to ensure a uniform study group. Hence, though the study group is small, its strength is that it is homogenous in terms of the disease process and the medical team practices and hence provides valuable information, which was utilized in the organization of the transition services.

## 5. Conclusion

This study provided us with valuable input from the patients who had experienced both the pediatric and adult care in the recent past and were in a unique position to provide genuine and workable solutions towards the establishment of a successful transition process. Implementation of these simple strategies has a global application for children with chronic disease transitioning to the adult services and will go a long way in ensuring safety and good outcomes for young adults.

## Figures and Tables

**Table 1:** What can be done to make the transition to adult care easier?

Suggestion	Parents *n* = 19	Patients *n* = 12	Total *n* = 31
Educate about their disease	14	4	18
Educate about insurance	13	4	17
Educate about college with transplant	13	3	16
Educate about drugs/alcohol/sex	13	4	17
Educate about medication	14	4	18
Educate about nutrition after transplant	12	3	15
Educate about total body care (hygiene, yearly exams, etc.)	13	7	20
Educate about support system	13	4	17
Make the kids listen	1	1	2
Give specific instructions about new place	0	1	1
Meet other transitioned teens	1	0	1
Teen clinic	1	0	1
Nothing	4	3	7

**Table 2:** How can parents be prepared and help prepare patients for the transition?

Suggestion	Parents *n* = 19	Patients *n* = 12	Total *n* = 31
Parent support Groups	16	3	19
Parents setting boundaries with children with chronic illness	2	1	3
Parents/caregivers letting kids grow and do self-care	8	5	13
Parents should not be involved in care	0	4	4
Review how adult care center works	1	0	1
Parents need to be more involved	2	2	4

**Table 3:** What should be changed to help with transition of care?

Suggestion	Parents *n* = 19	Patients *n* = 12	Total *n* = 31
Earlier education about facility	10	4	14
Let kids learn and not remind them so often	4	6	10
Educate about insurance and what to do if you lose it	1	1	2
Educate about how to deal with pregnancy	1	0	1
Nothing	1	2	3
